# Novel candidate biomarkers of origin recognition complex 1, 5 and 6 for survival surveillance in patients with hepatocellular carcinoma

**DOI:** 10.7150/jca.39163

**Published:** 2020-01-20

**Authors:** Xiang-Kun Wang, Qiao-Qi Wang, Jian-Lu Huang, Lin-Bo Zhang, Xin Zhou, Jun-Qi Liu, Zi-Jun Chen, Xi-Wen Liao, Rui Huang, Cheng-Kun Yang, Guang-Zhi Zhu, Chuang-Ye Han, Xin-Ping Ye, Tao Peng

**Affiliations:** 1 Department of Hepatobiliary Surgery, The First Affiliated Hospital of Guangxi Medical University, Nanning, 530021, Guangxi Province, China.; 2 Department of Medical Cosmetology, The Second Affiliated Hospital of Guangxi Medical University, Nanning 530000, Guangxi Province, China.; 3 Department of Hepatobiliary Surgery, The Third Affiliated Hospital of Guangxi Medical University, Nanning 530031, Guangxi Province, China.; 4 Department of Health Management and Division of Physical Examination, The First Affiliated Hospital of Guangxi Medical University, Nanning, 530021, Guangxi Zhuang Autonomous Region, People's Republic of China.; 5 Department of Hematology, The First Affiliated Hospital of Guangxi Medical University, Nanning, 530021, Guangxi Province, China.

**Keywords:** origin recognition complex, ORC1, ORC5, ORC6, biomarker, hepatocellular carcinoma.

## Abstract

**Background**: Hepatocellular carcinoma (HCC) has high morbidity and mortality and lacks effective biomarkers for early diagnosis and survival surveillance. Origin recognition complex (ORC), consisting of ORC1-6 isoforms, was examined to assess the potential significance of ORC isoforms for HCC prognosis.

**Methods**: Oncomine and Gene Expression Profiling Interactive Analysis (GEPIA) databases were used to examine differential isoform expression, stage-specific expression, calculate Pearson correlations and perform survival analysis. A human protein atlas database was utilized to evaluate the protein expression of ORCs in liver tissue. The cBioPortal database was used to assess isoform mutations and the survival significance of ORCs in HCC. Cytoscape software was employed to construct gene ontologies, metabolic pathways and gene-gene interaction networks.

**Results**: Differential expression analysis indicated that ORC1 and ORC3-6 were highly expressed in tumor tissues in the Oncomine and GEPIA databases, while ORC2 was not. All the ORCs were showed positive and statistically significant correlations with each other (all P<0.001). ORC1-2 and ORC4-6 expressions were associated with disease stages I-IV (all P<0.05), but ORC3 was not. Survival analysis found that ORC1 and ORC4-6 expressions were associated with overall survival (OS), and ORC1-3 and ORC5-6 expression were associated with recurrence-free survival (RFS; all P<0.05). In addition, low expression of these ORC genes consistently indicated better prognosis compared with high expression. Protein expression analysis revealed that ORC1 and ORC3-6 were expressed in normal liver tissues, whereas ORC2 was not. Enrichment analysis indicated that ORCs were associated with DNA metabolic process, sequence-specific DNA binding and were involved in DNA replication, cell cycle, E2F-enabled inhibition of pre-replication complex formation and G1/S transition.

**Conclusions**: Differentially expressed ORC1, 5 and 6 are candidate biomarkers for survival prediction and recurrence surveillance in HCC.

## Introduction

Liver cancer is one of the most common cancers worldwide. Hepatocellular carcinoma (HCC) is the most frequent type, comprising roughly 70% to 85% of all primary liver cancers 1. HCC ranks as the sixth most common malignancy in the world and accounts for approximately 7% of all malignancies 2. HCC is diagnosed in more than 740 000 new cases each year and is the third leading cause of malignancy-related deaths, with increasing incidence each year 3. Many risk factors have been identified for HCC, including chronic hepatitis B or C virus infection, type 2 diabetes mellitus, alcohol ingestion and metabolic syndrome, among others 4. Although these factors are well known, the prevention and surveillance of HCC is still burdensome and the prognosis remains unsatisfactory. Even with early diagnosis of HCC, it is difficult to treat advanced stage HCC due to its extra- and intrahepatic characteristics, including metastatic potential 5 and resistance to chemotherapeutic approaches such as hepatic artery infusion chemoembolization 6, transcatheter arterial chemoembolization 7 and the drug sorafenib 8. To improve the rate of early diagnosis of HCC and monitor recurrence after hepatectomy, many efforts have been made to develop new biomarkers for early diagnosis and recurrence prediction, including osteopontin, midkine, golgi protein-73 and α-fetoprotein-L3 9, but almost none of which have been widely accepted for clinical application.

Origin recognition complex (ORC), encoded by the latheo gene, is involved in the initiation of DNA replication and is composed of six isoforms, *ORC1-6* 10, 11. The ORC1 gene, also called HsOrc1 in humans, is weakly expressed in quiescent cells, but can be upregulated by cell growth signals 11. The transcription of ORC1 is dependent on the E2F transcription factor 11. In contrast, ORC2 expression is regulated differently from ORC1 11. Cohen et al. examined the localization of ORC1 in human leukemia cells and found that it was localized to the pericentriolar region during anaphase 12. Phosphorylation of ORC2 and HBO1 induced by polo-like kinase 1 leads to gemcitabine resistance in pancreatic cancer 13. Phosphorylation of ORC2 by polo-like kinase 1 promotes DNA replication under stress conditions 14. Knockdown of ORC3 increased the level of mRNP-bound Nxf1, while knockdown of ORC5 decreased the association between Nxf1 and mRNP 15. ORC6 is crucial for the formation of pre-replicative complex interactions with ORC chaperone proteins that promote chromatin binding of ORC 16.

ORC and the mini-chromosome maintenance (mcm) complex are well known to be involved in DNA biosynthesis in the cell cycle pathway. Our previous results indicated that the MCM complex is not only associated with the overall survival (OS) of HCC, but can also serve as both a diagnostic and prognostic biomarker for HCC 17. Therefore, we conducted the present study to explore potential significance of ORC in HCC.

## Material and methods

### Oncomine database analysis

The Oncomine database (https://www.oncomine.org/resource/main.html) was first used to profile ORC1-6 isoform expression in multiple cancer types, including liver cancer, bladder cancer, breast cancer, cervical cancer, colorectal cancer, gastric cancer and esophageal cancer. The Oncomine database was then used to compare ORC1-6 isoform expression in HCC and normal liver tissues in several datasets, including the Chen liver 18, Roessler liver and Roessler liver 2 19, Mas liver 20, Wurmbach liver 21 and Guichard liver 22 datasets. Criteria for the inclusion and exclusion: all the enrolled objects were surgical resection and pathological diagnosed of HCC.

### Gene Expression Profiling Interactive Analysis (GEPIA) database analysis

The GEPIA2 database (http://gepia2.cancer-pku.cn/#index) was used to explore transcripts per million and further determine ORC1-6 isoform expression in HCC and normal liver tissues 23. Survival analysis, including OS and disease-free survival (DFS), was employed to examine the prognostic significance of ORC1-6 isoforms in HCC at median cutoff. In addition, Pearson correlation analysis identified correlations between each two ORC isoforms in tumor tissues, and analysis by stage determined the associations between ORC isoform expression and disease stage.

### Human protein atlas (HPA) and cBioPortal database analysis

The HPA database (http://www.proteinatlas.org/) was utilized to identify protein expression of ORC isoforms in liver tissue in a tissue-based map of the human genome 24. Brown color indicated higher expression compared with nattier blue. The cellular location of the ORCs was identified using the HPA database with a subcellular map 25. Meanwhile, the cBioPortal database (http://www.cbioportal.org/) was used to identify the types of ORC isoform mutations, including mutation, amplification, and deep deletion, and to determine prognosis significance of isoforms in the presence and absence of mutations 26, 27. An interaction network between ORC isoforms and other genes was constructed using the cBioPortal database as well.

### Gene ontology (GO), KEGG pathway enrichment analysis and interaction network construction

ORC1-6 isoforms underwent enrichment analysis of GO terms and Kyoto Encyclopedia of Genes and Genomes (KEGG) pathways. GO terms, including biological processes (BPs), cellular components (CCs) and molecular functions (MFs), were enriched and visualized using the BinGO plugin in Cytoscape software 28, 29. KEGG pathways were enriched and visualized using the ClueGO plugin in Cytoscape software 30. Gene-gene interaction and protein-protein interaction networks were constructed using the geneMANIA plugin in Cytoscape software 31 and the STRING website (https://string-db.org/) 32.

### Statistical analysis

Gene expression analysis by stage and differential analysis utilized one-way analysis of variance. Survival analysis was performed using the log-rank test and Cox test. Cox proportional hazard ratios and 95% confidence intervals were included in the survival plots. A p-value ≤ 0.05 was considered statistically significant.

## Results

### Gene expression analysis

**Figure 1:**
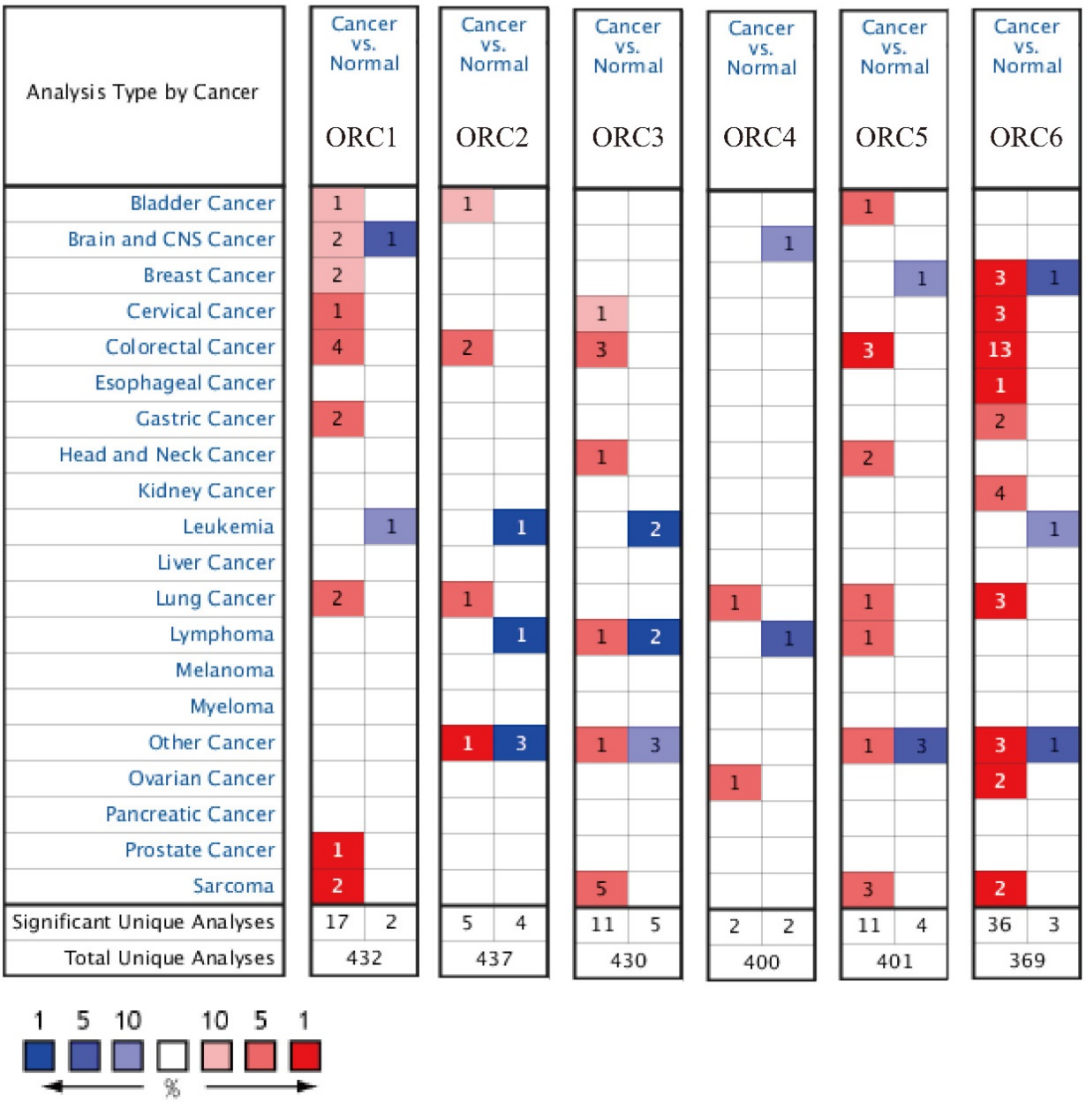
Analysis of ORC1-6 isoforms in multiple cancers.

**Figure 2:**
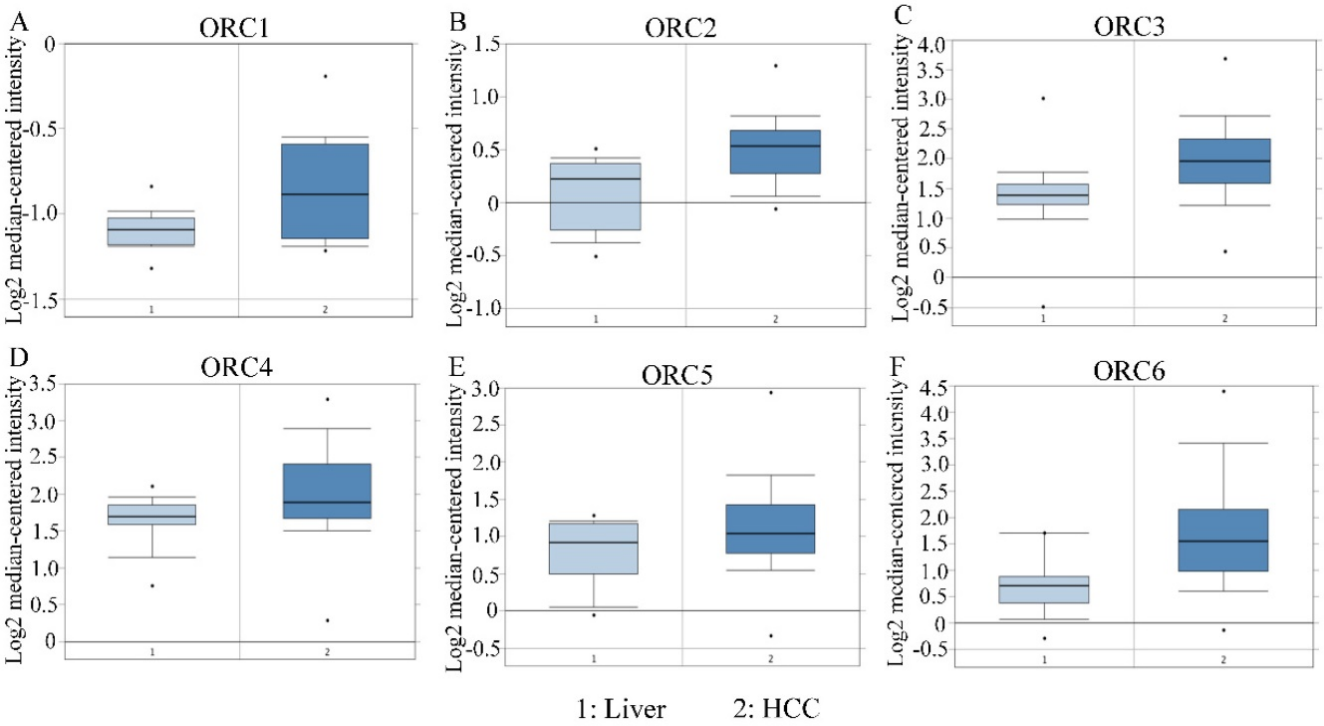
Expression of ORC1-6 isoforms in normal liver and HCC tissues. A-F: Expression of ORC1-6 isoforms in normal liver and HCC tissues.

**Figure 3:**
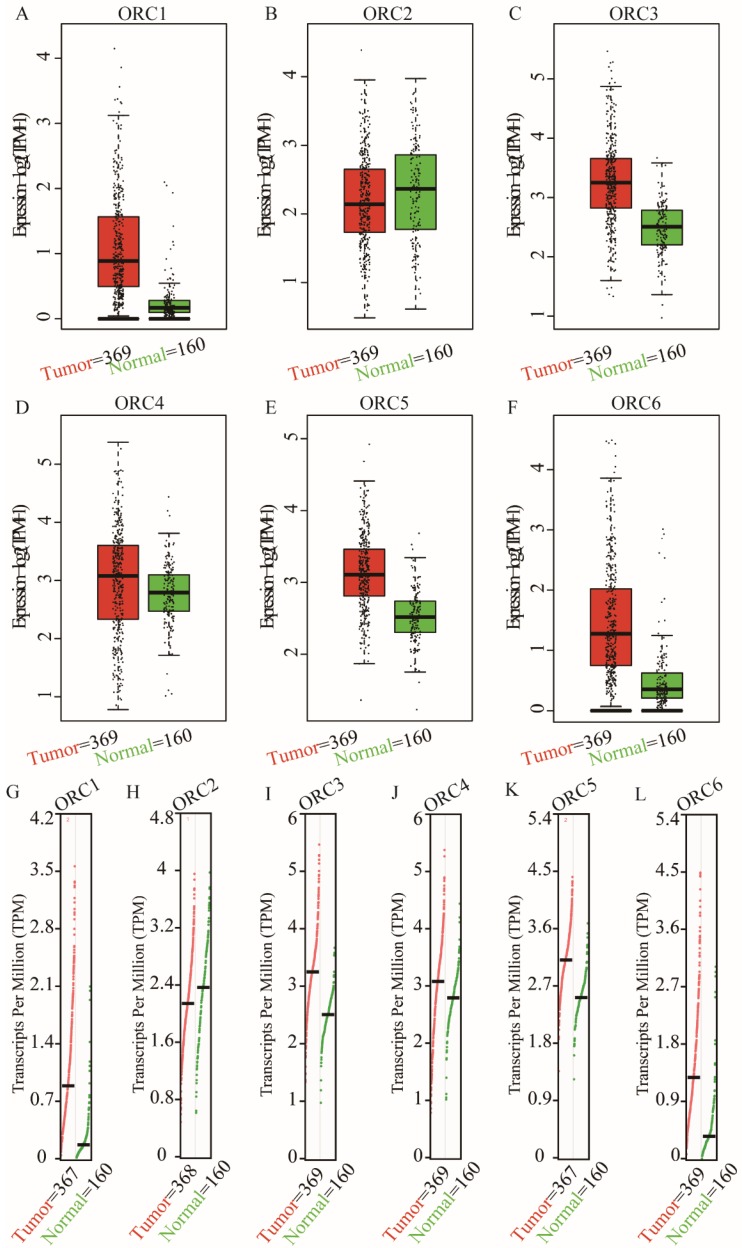
Expression and transcripts of ORC1-6 isoforms in normal liver and tumor tissues. A-F: Expression of ORC1-6 isoforms in normal liver and tumor tissues; G-L: Transcripts of ORC1-6 isoforms in normal liver and tumor tissues.

Studies about ORC isoforms in multiple cancers are shown in Figure 1. The significance of ORCs has been explored in many types of cancers, especially breast cancer, colorectal cancer, leukemia and lung cancer (Figure 1). The expression levels of ORC isoforms in HCC and normal liver tissues are shown in Figure 2. All isoforms were highly expressed in HCC tissues compared with normal liver tissues (Figure 2A-F). Expression level changes and the p-values of changes in ORCs were subsequently analyzed. Detailed changes between HCC and normal liver tissues in multiple datasets are presented in Table 1. Validation studies of the expression levels of ORCs in HCC were performed in the GEPIA database, which indicated that ORC1 and ORC3-6 presented high levels of expression in tumor tissues, while ORC2 showed the opposite results (Figure 3).

### Protein expression and cellular locations

**Figure 4:**
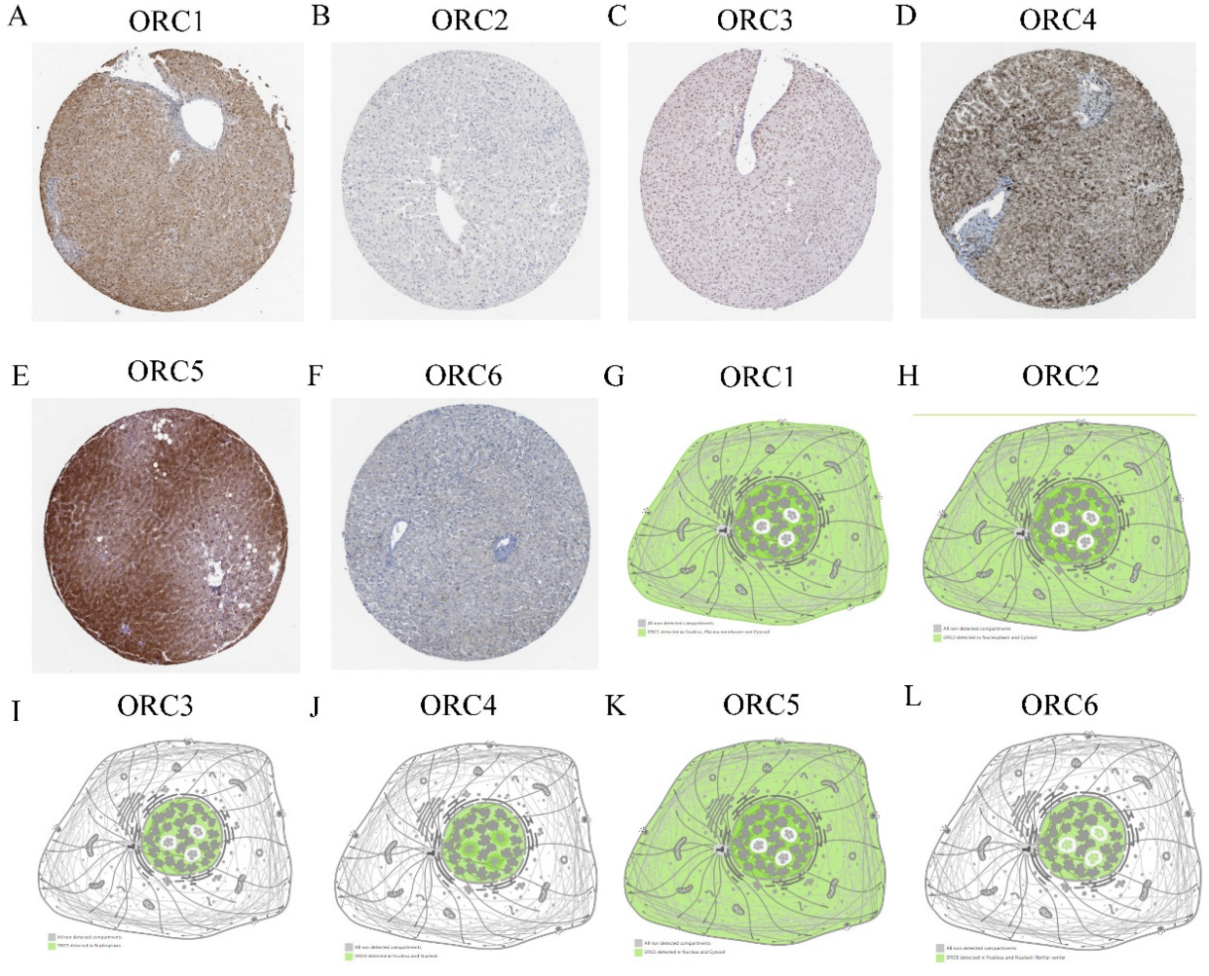
Protein expression and localization of ORC1-6 isoforms in liver tissue and cell lines. A-F: Protein expression of ORC1-6 isoforms in liver tissues; G-L: Localization of ORC1-6 isoforms in cell lines.

Protein expression and cellular locations of ORCs were analyzed in the HPA database. Protein expression analysis found that ORC1 and ORC3-5 showed moderately positive expression in normal liver tissues (Figure 4A, C-E); ORC6 showed weakly positive expression (Figure 4F), whereas ORC2 was not expressed in liver tissue (Figure 4B). ORC1 was detected in the nucleus, plasma membrane and cytosol (Figure 4G); ORC2 was detected in nucleoplasm and cytosol (Figure 4H); ORC3 was detected in nucleoplasm (Figure 4I); ORC4 was detected in the nucleus and nucleoli (Figure 4J); ORC5 was detected in nucleus and cytosol (Figure 4K); and ORC6 was detected in the nucleus and nucleoli fibrillar center (Figure 4L).

### OS and DFS analysis

**Figure 5:**
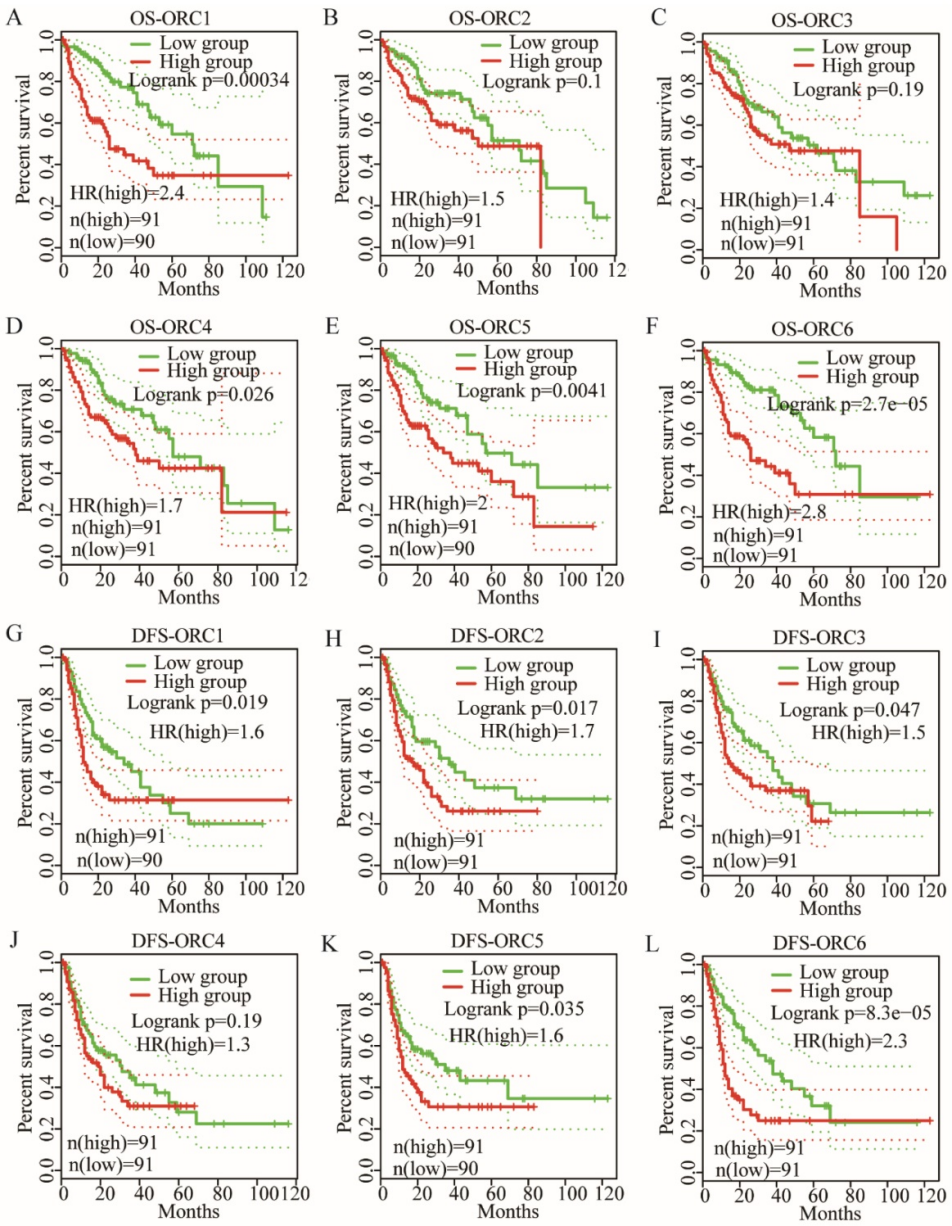
OS and RFS plots of patients expressing ORC1-6 isoforms in HCC. A-F: OS plots of patients expressing each of the ORC1-6 isoforms in HCC; G-L: RFS plots of patients expressing each of the ORC1-6 isoforms in HCC.

OS analysis indicated that ORC1 and ORC4-6 expression were associated with prognosis (Log-rank test results: P<0.001, P=0.026, P=0.004, and P<0.001 for Figure 5A, D-F, respectively), whereas ORC2 and ORC3 were not associated with prognosis (Log-rank test results: P=0.1 and P=0.19 for Figure 5B-C, respectively). Moreover, low expression of ORC1 and ORC4-6 were consistently associated with better prognosis compared with higher expression.

DFS analysis indicated that ORC1-3 and ORC5-6 expression were associated with prognosis (Log-rank test results: P=0.019, P=0.017, P=0.047, P=0.035 and P<0.001, respectively, for Figure 5G-I, K-L), but ORC4 expression was not associated with prognosis (Log-rank test, P=0.1; Figure 5J). Moreover, all five ORCs consistently revealed that low expression was associated with better prognosis compared with high expression. These results suggested that ORC isoforms may act as oncogenes in HCC.

### Pearson correlation and stage analysis

**Figure 6:**
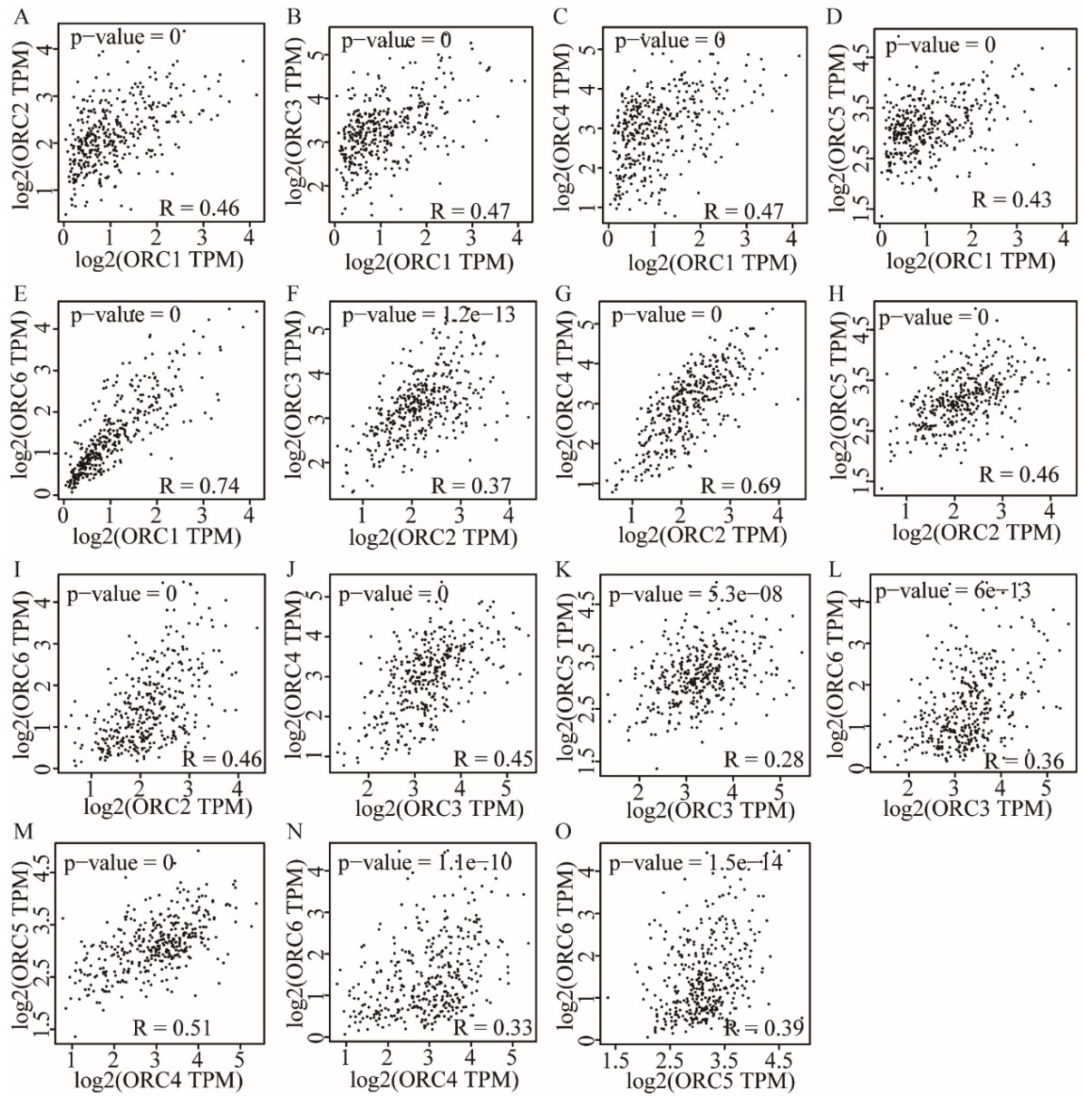
Pearson correlation plots among ORC1-6 isoforms in HCC tissues. A-E: Pearson correlation plots between ORC1 and ORC2-6 isoforms; F-I: Pearson correlation plots between ORC2 and ORC3-6 isoforms; J-L: Pearson correlation plots between ORC3 and ORC4-6 isoforms; M-N: Pearson correlation plots between ORC4 and ORC5-6 isoforms; O: Pearson correlation plots between ORC5 and ORC6.

**Figure 7:**
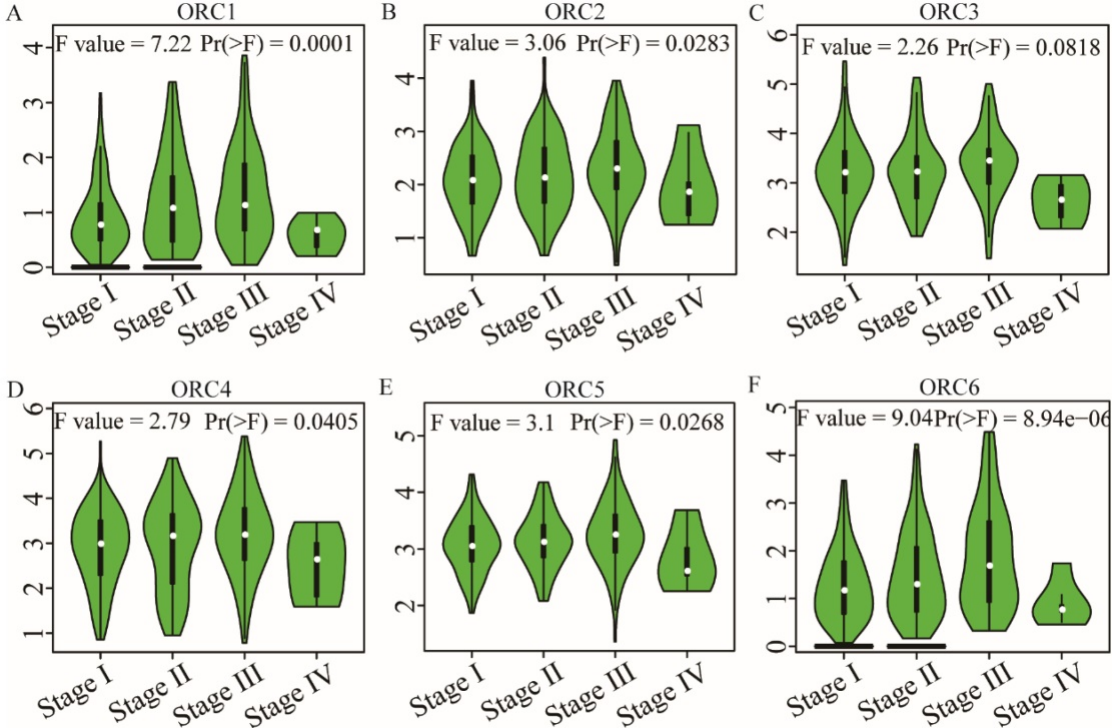
Analysis of the expression of ORC1-6 isoforms at different disease stages. A-F: Analysis of ORC1-6 isoform expression at each disease stage.

Pearson correlation analysis was performed for ORC isoforms in HCC samples. Analysis findings demonstrated that each two ORC isoforms were positively correlated (Figure 6). In addition, all correlations were statistically significant (P<0.05). Furthermore, analysis by disease stage found that all ORCs exhibited higher expression with progression from stage I to stage III (Figure 7). However, stage IV always induced the lowest expression compared with stages I, II and III for all the ORCs. To summarize, ORC3 was not significantly different among stages I-IV (P=0.0818, Figure 7C), but the other isoforms were significantly associated with stages I-IV (P=0.0001, P=0.00283, P=0.0405, P=0.0268 and P=8.94e-6, respectively; Figure 7A-B, D-F).

### Mutation analysis and interaction network

**Figure 8:**
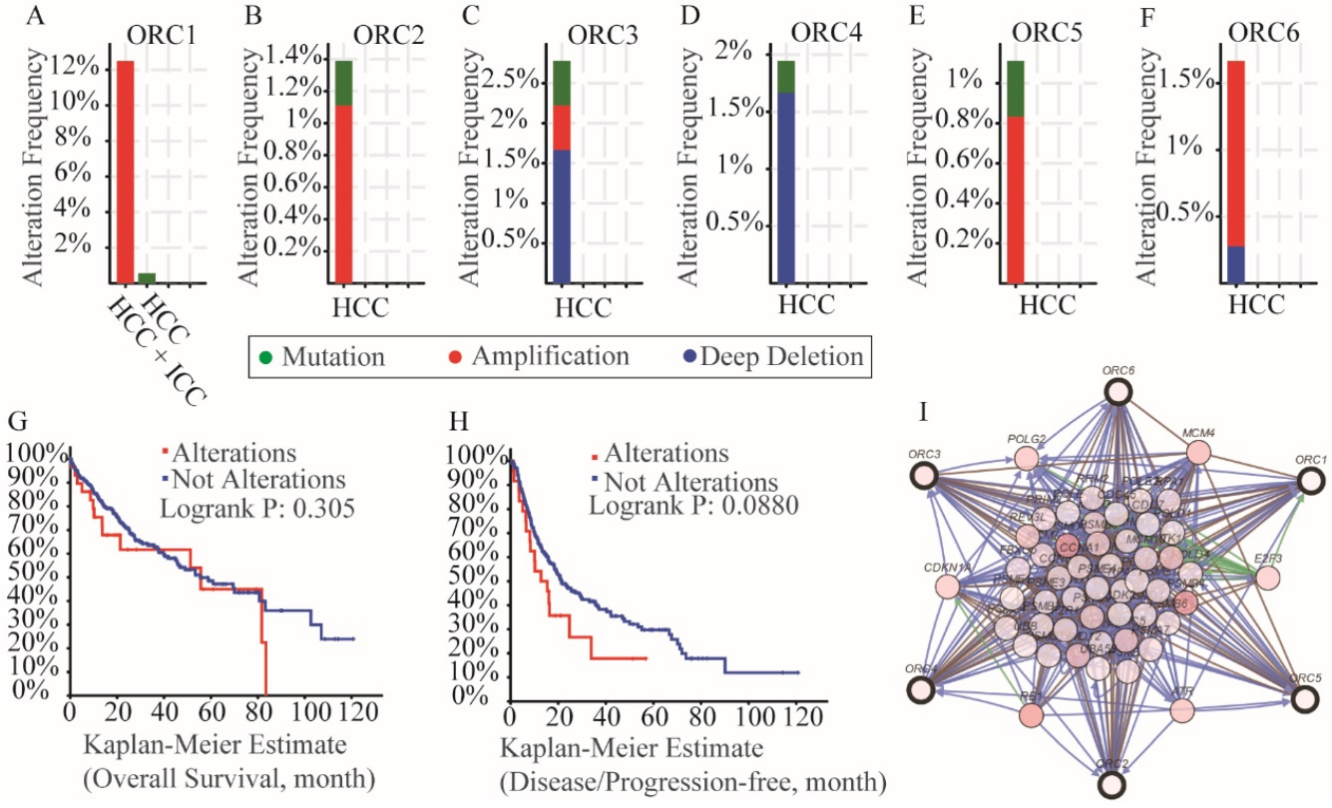
Analysis of mutations, survival plots and interaction networks of ORC1-6 isoforms in HCC and ICC. A-F: Analysis of mutations of ORC1-6 isoforms in HCC and ICC; G-H: OS and RFS in patients expressing ORC1-6 isoform mutations; I: Interaction network among ORC1-6 isoforms.

Mutation analysis in the cBioPortal database indicated that all of the ORCs had mutations (Figure 8A-F). Specifically, ORC1 had amplification more than 12% in HCC and intrahepatic cholangiocarcinoma (ICC) and mutation less than 2% HCCs (Figure 8A); ORC2 had amplification more than 1% and mutation less than 1% (Figure 8B); ORC3 had amplification, mutation and deep deletion more than 2.5% together (Figure 8C); ORC4 had mutation and deep deletion more than 1.5% together (Figure 8D); ORC5 had mutation and amplification more than 1% together (Figure 8E); ORC6 had amplification and deep deletion more than 1.5% together (Figure 8F). In addition, survival analysis including OS and DFS with and without mutations of ORC isoforms suggested that mutations did not associate with either OS or DFS (Figure 8G-H). We then constructed an interaction network using the ORC isoforms, which demonstrated that ORCs acted in concert with PSM family members such as PSME1, PSMB6 and PSMC4 to control expression of RRM2, POLE, POLE2, E2F3 and TK1, and showed controlling state changes of REV3L, CDC7, MCM7, ATR, PSMB3 and others (Figure 8I).

### Enrichment analysis and interaction network of gene-gene and protein-protein interactions

**Figure 9:**
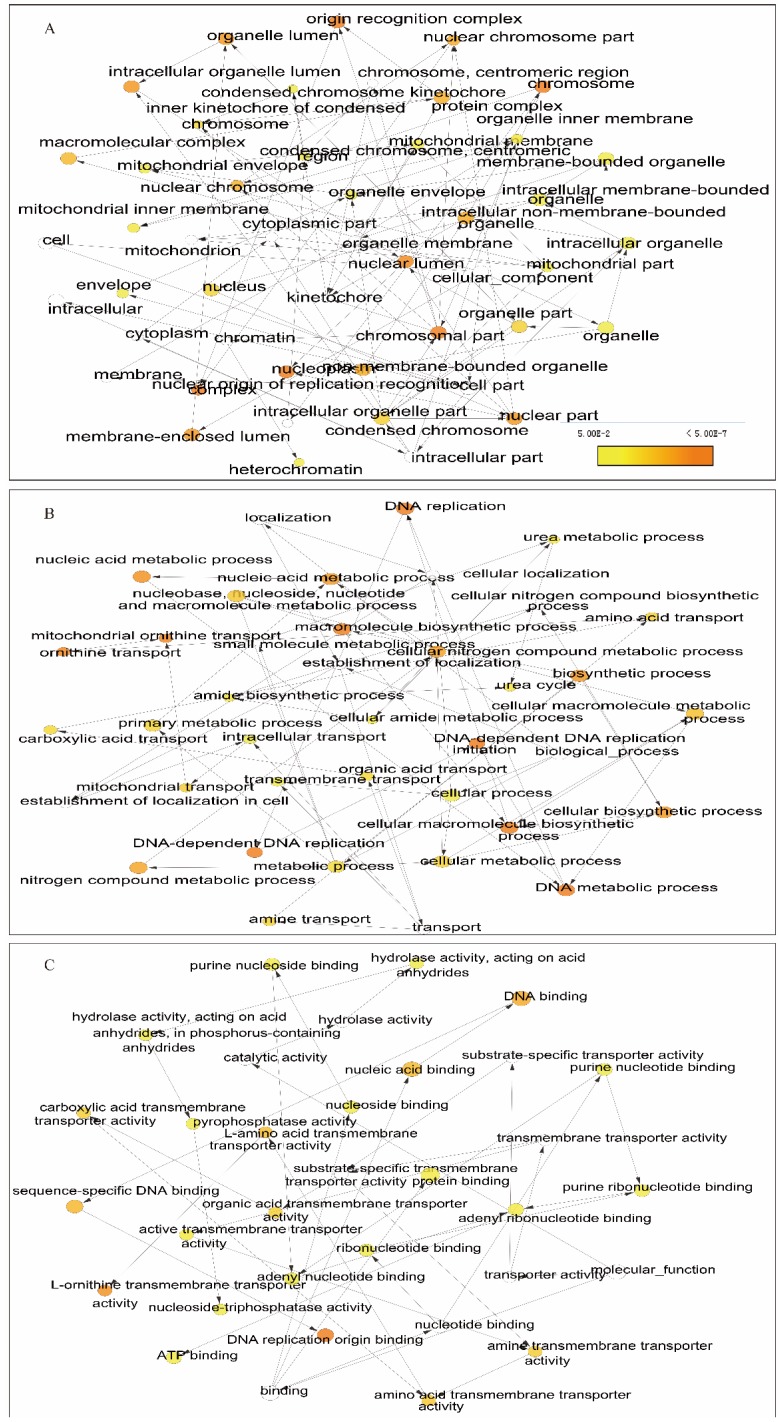
Enriched biological processes, cellular components and molecular functions networks of ORC1-6 isoforms. A-C: Networks of ORC1-6 isoforms showing enriched cellular components (A), biological processes (B) and molecular functions (C).

**Figure 10:**
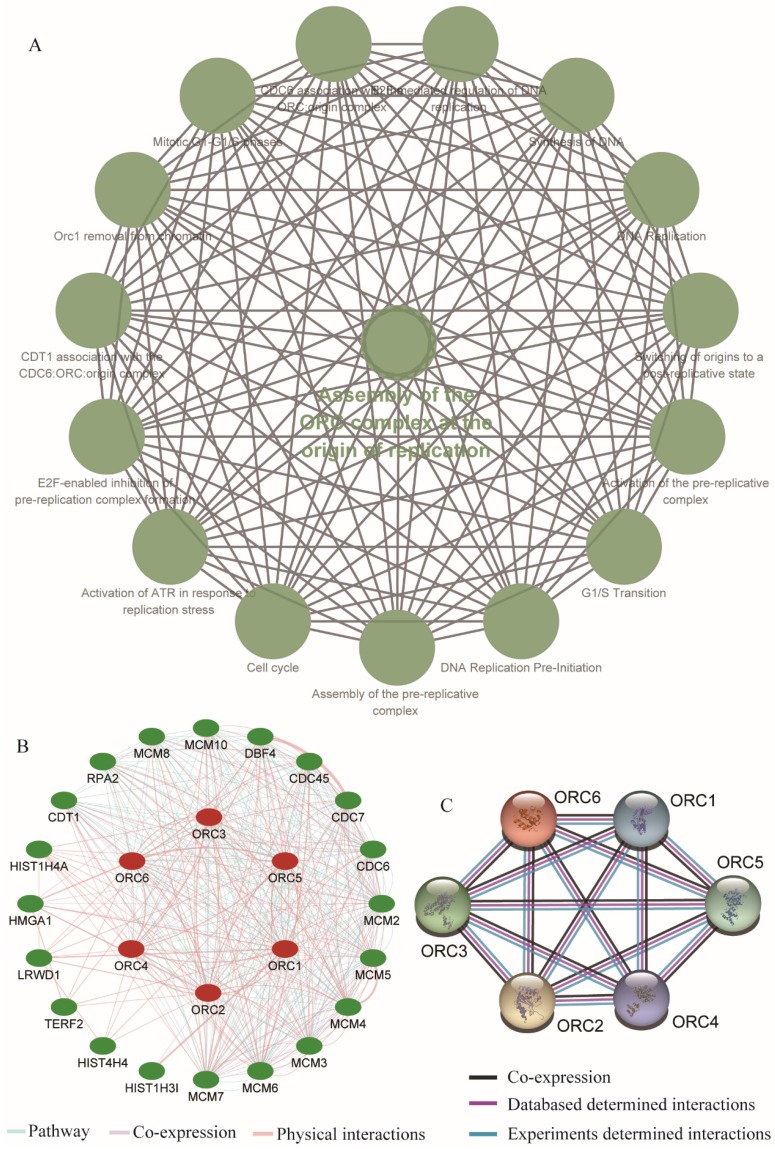
Enriched KEGG pathways, gene-gene and protein-protein interaction networks of ORC1-6 isoforms. A: Enriched KEGG pathways of ORC1-6 isoforms; B: Gene-gene interaction network of ORC1-6 isoforms; C: Protein-protein interaction network of ORC1-6 isoforms.

We further explored metabolic pathways and GO terms of ORCs. Enrichment of GO terms indicated that ORCs were involved in chromosomal part, the nuclear origin of ORC, the nuclear lumen and macromolecular complexes, etc. in CCs (Figure 9A); DNA-dependent DNA replication, DNA metabolic processes and cellular biosynthetic processes, etc. in BPs (Figure 9B); DNA binding, sequence-specific DNA binding and L-ornithine transmembrane transporter activity, etc. in MFs (Figure 9C). Pathway results indicated that ORCs were involved in DNA replication, cell cycle, E2F-enabled inhibition of pre-replication complex formation and the G1/S transition, etc. (Figure 10A). The gene-gene interaction network suggested that ORCs were co-expressed interactively with MCM2-8, MCM-10, CDC6-7 and CDC45, etc. (Figure 10B). ORCs showed interactions in the pathway with MCM members and CDC members. The protein-protein interaction network suggested that all of the ORCs were co-expressed and have determined interactions in experiments and databases (Figure 10C).

## Discussion

Our present study explored the potential prognostic significance of ORC1-6 isoforms in HCC. We discovered that ORC1-6 were highly expressed in HCC tissues compared to normal liver tissues. In addition, we found that ORC1 and ORC4-6 expression were associated with OS and that ORC1-3 and ORC5-6 expressions were associated with DFS. Low expression of these genes consistently indicated better prognosis compared with higher expression. These results suggest that ORC isoforms may act as oncogenes in HCC. Protein expression analysis revealed that ORC1 and ORC3-6 were also expressed in normal liver tissues. Combining ORC expression and survival analyses, we conclude that ORC1, 5 and 6 are candidate biomarkers for survival prediction and recurrence surveillance. Enrichment analysis indicated that ORCs were associated with DNA replication, chromosomal part, DNA metabolic process, DNA binding and sequence-specific DNA binding, and were also involved in DNA replication, cell cycle, E2F-enabled inhibition of pre-replication complex formation and the G1/S transition. Further investigation to validate the above findings is warranted.

ORC, consisting of six subunits, functions as the initiator in recognizing replication start sites and interacts with subsequent replication factors 33. It was first discovered in *Saccharomyces cerevisiae* as a multi-protein complex that was connected to the autonomously replicating sequence 34. The ORC plays a pivotal role in the initiation of DNA replication in all eukaryotic systems 16. Molecular interactions within the eukaryotic replication origin occur within the overall structure 12. In addition, melting of the DNA double helix facilitates a conformational change in the ORC-related post-replicative state which can prevent the re-initiation of replication at the specific binding site 35. In eukaryotes, ORC is associated with chromatin at multiple sites 36-38 and these sites may be candidate replication origins, which initiate the assembly of the pre-replication complex at the G1 phase of the cell cycle 39, 40.

In addition to its main role in the formation of the pre-replication complex on chromosomes prior to DNA replication, subunits of ORC have been reported to be involved in several chromosome-associated processes 33, 41. Hemerly et al. found that ORC1 controls centriole and centrosome re-duplication, as well as the initiation of DNA replication in human U2OS cells 42. ORC2 and other ORC isoforms were first reported as essential for DNA replication initiation by connecting to replication origin sites to form the pre-replication complex at late G1 and early S phase in mammalian cells 41. ORC2 was found localized to the centrosome and centromere, which was essential for proper chromatin segregation at the G2/M phase 43. Wang et al. found that ORC2 was modified by the small ubiquitin-like modifier (SUMO) at the G2/M phase of the cell cycle and that SUMOylation of ORC2 was crucial for the smooth transition into mitosis 44. ORC2 also localized to the telomeric region and plays a pivotal role in telomere homeostasis 45, 46. Depletion of ORC2 leads to mitotic arrest because of defects in chromosome condensation 43.

ORC3 interacts with HP1 at the heterochromatin foci to promote the organization of higher chromatin structure 47. ORC4 and ORC6 can directly interact with ENY2, which is bound to type C2H2 zinc fingers of insulator protein Su (Hw) and CTCF in *Drosophila* 48-50. Moreover, protein Su (Hw) opens chromatin regions and accelerates the recruiting of the ORC to chromatin 51. ORC5 interacts with the histone acetyltransferase GCN5/KAT2A, making origins of replication more accessible for activation 47. Multi-mono-ubiquitylation of ORC5 facilitates this kind of interaction, enabling opening of the local origin chromatin environment and stimulating origin activation 52.

ORC6 is the most divergent and evolutionarily least conserved subunit among all the ORC proteins 16. ORC6 interacts with other chaperone proteins, such as high mobility group protein A1a, which may be helpful for targeting ORCs to specific chromatin regions in addition to functioning in the assembly of the pre-replicative complex 53. ORC6 binds to the outer kinetochore during mitosis and localizes to the midplane of cell division in anaphase where it is required for cytokines through a connection with septin protein 16. Thomae et al. found that replication competence was significantly associated with the potential of mutant forms to cooperate with human ORCs, suggesting an active role of ORC6 in origin activation 16. They also observed that ORC6 was abundantly expressed in correlation with ORC2 expression and contributes to the replication of the initiation process independent from ORC1-5 16.

Diffley et al. reported that the main function of ORCs was to initiate replication origins in the G1 phase of the cell cycle 39, 40. However, DePamphilis demonstrated that ORCs were related to chromatin at other stages of the cell cycle or in G0 cells 54. These findings suggest that the functions of ORCs are not restricted to DNA replication. Sasaki et al. demonstrated that ORC also initiated non-replication functions 55, 56 such as transcription silencing 57, chromatid cohesion 58, neuron development 59 and cytokinesis 60. Notably, previous studies have reported the association of ORC with human diseases, such as Meier-Gorlin syndrome, Epstein-Barr virus-infected diseases, American trypanosomiasis and African trypanosomiasis 55.

More specifically, De Munnik et al. observed mutations of ORC1, 4 and 6 in a study involving 35 individuals with Meier-Gorlin syndrome 61. Young indicated that because ORC1 was significantly upregulated after irradiation for 6 and 24 hours in PC-3 cells, ORC1 and other DNA repair candidates may be potential targets for radiation sensitization and serve as predictive biomarkers for prostate cancer 62. Chen et al. found that ORC1 participated in the XIST/miR-140-5p/ORC1 axis of progression in cervical cancer, which will shed new light on epigenetic diagnostics and therapeutics in cervical cancer 63. Our present study found that ORC1 was involved in DNA binding and biosynthesis in the cell cycle, which is consistent with previous findings of the main functions of ORCs 39, 40, 54. Moreover, our study indicated that ORC1 was not only associated with both OS and DFS, but was also a potential biomarker for HCC survival prediction and recurrence surveillance. This potential role of ORC1 in tumors is in accordance with previous reports of cervical cancer and prostate cancer 62, 63.

A study by Radojkovic et al. found a novel mutation A286V within ORC4 and reported that this mutation might be a favorable prognostic biomarker for B-cell lymphoproliferative disorders 64. Our study revealed that ORC4 expression, highly expressed in tumor tissue, was associated with OS and might be a candidate biomarker for HCC. ORC2 and ORC5 were reported to be upregulated in microarray data originating from the cisplatin-sensitive T24 cell line and cisplatin-resistant T24R2 cell line 65. However, this study did not report any prognostic value for ORC2 and ORC5. Our study indicated that ORC2 expression was correlated with DSF in HCC; furthermore, ORC5 expression was associated with both OS and DFS in HCC, suggesting it is a novel candidate biomarker for HCC survival. The role of ORC3 has not yet been documented in tumors. However, a single nucleotide polymorphism of ORC6 was reported as a breast cancer-related candidate gene 66. Xi et al. found that ORC6 was highly expressed in tumors compared to paired normal tissues in colorectal cancer 67. Thus, our results agree with Xi et al.'s finding of high expression of ORC6 in tumor tissues. These authors further demonstrated that ORC6 expression was associated with 5-fluorouracil-related resistance in human colorectal cell lines 68. Moreover, they found that decreased ORC6 expression sensitized human colorectal cell lines to 5-fluorouracil and cisplatin, and thus, ORC6 may be a novel therapeutic target in colon cancer 69. Our present study demonstrated that ORC6 was correlated with both OS and DFS of HCC, indicating that it may serve as a prognostic biomarker for HCC survival.

There are several limitations of our study, which should be recognized. First, our findings will require further large population and cohort validations, including clinical parameters and our medical center cohort validation. Second, our study results need to be further validated in Asian population cohorts, including our medical center cohort. Then, additional experiments are needed to explore the specific mechanisms underlying ORC involvement in HCC. Finally, the potential clinical application of ORCs should be explored in multi-race studies in multiple countries.

## Figures and Tables

**Table 1:** Analysis of ORC isoform expressions in HCC and NR.

Gene	Reporter	Types	FC	P value	T test	Reference
ORC1	IMAGE:194236	HCC vs. NR	1.477	1.34E-4	3.724	Chen liver (^Reference 55^)
	205085 _at	HCC vs. NR	1.156	0.001	3.287	Roessler liver (^Reference 56^)
	205085 _at	HCC vs. NR	1.133	3.21E-9	5.933	Roessler liver 2 (^Reference 56^)
	205085 _at	HCC vs. NR	1.052	0.021	2.109	Mas liver (^Reference 57^)
	205085 _at	HCC vs. NR	1.271	0.007	2.613	Wurmbach liver (^Reference 58^)
ORC2	204853 _at	HCC vs. NR	1.322	2.78E-5	4.620	Mas liver (^Reference 57^)
ORC3	IMAGE:260336	HCC vs. NR	1.789	6.71E-14	8.037	Chen liver (^Reference 55^)
	210028_s_at	HCC vs. NR	1.511	1.80E-31	12.701	Roessler liver 2 (^Reference 56^)
	210028_s_at	HCC vs. NR	1.249	3.77E-4	3.609	Mas liver (^Reference 57^)
	210028_s_at	HCC vs. NR	1.316	0.002	3.083	Roessler liver (^Reference 56^)
ORC4	203351_s_at	HCC vs. NR	1.366	2.25E-19	9.367	Roessler liver 2 (^Reference 56^)
	203351_s_at	HCC vs. NR	1.287	0.011	2.417	Roessler liver (^Reference 56^)
	203352 _at	HCC vs. NR	1.082	1.22E-5	4.268	Roessler liver 2(^Reference 56^)
ORC5	204957 _at	HCC vs. NR	1.445	2.97E-22	10.185	Roessler liver 2 (^Reference 56^)
	211212_at	HCC vs. NR	1.217	2.02E-13	7.513	Roessler liver 2 (^Reference 56^)
	07-103594861	HCC vs. NR	1.053	1.71E-7	5.439	Gurchard liver (^Reference 59^)
	204957 _at	HCC vs. NR	1.293	0.015	2.245	Roessler liver (^Reference 56^)
	211212_at	HCC vs. NR	1.132	0.042	1.797	Roessler liver (^Reference 56^)
	204957 _at	HCC vs. NR	1.275	0.009	2.575	Wurmbach liver (^Reference 58^)
ORC6	219105_x_at	HCC vs. NR	1.690	4.12E-5	4.728	Roessler liver (^Reference 56^)
	219105_x_at	HCC vs. NR	2.002	1.26E-6	4.166	Wurmbach liver (^Reference 58^)
	219105_x_at	HCC vs. NR	1.476	6.75E-23	10.623	Roessler liver 2 (^Reference 56^)

Note: HCC: hepatocellular carcinoma; NR: normal; FC: fold change; ORC1: origin recognition complex 1; ORC2: origin recognition complex 2; ORC3: origin recognition complex 3; ORC4: origin recognition complex 4; ORC5: origin recognition complex 5; ORC6: origin recognition complex 6.
